# Water-Soluble Humic Materials Regulate Quorum Sensing in *Sinorhizobium meliloti* Through a Novel Repressor of *expR*

**DOI:** 10.3389/fmicb.2018.03194

**Published:** 2018-12-21

**Authors:** Yuan-Yuan Xu, Jin-Shui Yang, Cong Liu, En-Tao Wang, Ruo-Nan Wang, Xiao-Qian Qiu, Bao-Zhen Li, Wen-Feng Chen, Hong-Li Yuan

**Affiliations:** ^1^State Key Laboratory of Agrobiotechnology and Key Laboratory of Soil Microbial, Ministry of Agriculture, College of Biological Sciences, China Agricultural University, Beijing, China; ^2^Escuela Nacional de Ciencias Biológicas, Instituto Politécnico Nacional, Mexico City, Mexico

**Keywords:** *Sinorhizobium meliloti*, quorum sensing, humic materials, ExpR regulator, bacterial communication

## Abstract

Quorum sensing (QS) plays an important role in the growth, nodulation, and nitrogen fixation of rhizobia. In this study, we show that water-soluble humic materials (WSHM) repress the expression of the QS related genes *sinI, sinR*, and *expR* in *Sinorhizobium meliloti.* This decreased the production of *N*-acetyl homoserine lactones (AHL) and exopolysaccharides (EPS), and ultimately increased *S. meliloti* cell density. We also identified a novel regulator, SMc03890 (renamed QsrR), which binds directly to the *expR* promoter. Deletion of *qsrR* increased *expR* expression. WSHM repressed the expression of *expR* by augmenting the interaction between QsrR and the *expR* promoter; this was determined by a bacterial-one-hybrid assay. These effects of WSHM on the QS system in *S. meliloti* may be the underlying mechanism by which WSHM increase the symbiotic nitrogen fixation of *Medicago sativa* inoculated with *S. meliloti*. This study provides the first evidence that humic acids regulate the QS of rhizobia and suggests that WSHM could be used as fertilizers to improve the efficiency of symbiotic nitrogen fixation.

## Introduction

**FIGURE 1:**
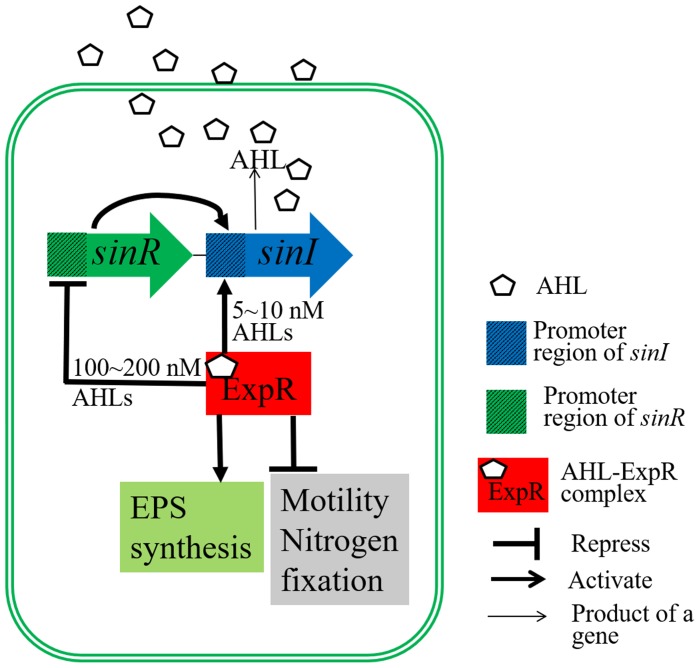
Regulatory diagram of the *S. meliloti* quorum sensing system. The transcription of *sinI*, which encodes AHL synthase, is induced by SinR. *sinI* transcription can also be induced by the AHL-ExpR complex at low AHL concentrations (5∼10 nM). The AHL-ExpR complex represses *sinR* expression at high AHL concentrations (100∼200 nM). Furthermore, the AHL-ExpR complex induces the expression of genes related to EPS biosynthesis, and represses the expression of genes important for motility, nodulation, and nitrogen fixation.

Quorum sensing (QS) is a bacterial communication mechanism in which cell physiology and behavior are coordinated with population density (Bogino et al., 2015). In symbiotic nitrogen-fixing bacteria (rhizobia), QS plays a key role in their growth and the formation of symbiosis with their legume hosts (Bogino et al., 2015; Koul et al., 2016). In the QS system of *Sinorhizobium meliloti* 8530 (Figure 1), SinI is responsible for synthesis of the QS signaling molecules, *N*-acetyl homoserine lactones (AHL). The expression of *sinI* is induced by SinR; meanwhile, ExpR either mediates a positive regulatory feedback loop by inducing the expression of *sinI* or a negative feedback regulation by down-regulating the expression of *sinR*, depending on the AHL concentration (Charoenpanich et al., 2013; Calatrava-Morales et al., 2018). In addition to regulating the genes involved in nodulation and nitrogen fixation (Hoang et al., 2004), AHL-ExpR complex also up-regulate the expression of *exp* operon, which is involved in EPS II synthesis, and down-regulate genes related to bacterial motility, such as *visN, visR*, and *rem* (Gurich and Gonzalez, 2009; Mueller and González, 2010). The number of pink nodules induced by the *sinI* mutant decreased compared with the WT strain due to the inability of the *sinI* mutant to repress the expression of motility genes at high cell densities (Gurich and Gonzalez, 2009). The *expR* mutant, *S. meliloti* 1021, grows more rapidly and has an increased nodule occupancy (10∼20% higher than the WT strain, *S. meliloti* 8530) (Charoenpanich et al., 2015).

Many compounds have been reported to interfere with the bacterial QS systems (Kalia, 2013; Gonzalez-Ortiz et al., 2014). For rhizobia, L-canavanine, which is extracted from alfalfa seed exudates, inhibits the expression of *S. meliloti expR* gene (Keshavan et al., 2005), while the flavonoids induce the expression of AHL synthesis genes in *Sinorhizobium fredii* SMH12 and in *Rhizobium etli* ISP42 (Pérez-Montaño et al., 2011). Generally, compounds that interfere with microbial QS systems are heterocyclic compounds containing groups such as furan, pyridine, butyrolactone, benzene ring, and quinoline (Gonzalez and Keshavan, 2006; Christensen et al., 2013). These compounds have chemical structures similar to those of humic materials (Gao T.G. et al., 2015); thus, it is possible that humic materials may also interfere with microbial QS systems.

Humic materials are supramolecules derived from the residues of degraded plant, animal and microbial cells (Hayes and Wilson, 1997), with structures of relatively small self-assembled molecules that are held together by multiple weak interactions, such as hydrogen and van der Waals bonds. Generally, humic materials include, but are not limited to, *n*-alkanoic acids, *n*-alkanols, hydrocarbons, hydroxyacids, aromatic compounds, polyhydroxylated compounds, steroids, terpenoids, and *N*-heterocyclic compounds (Nebbioso and Piccolo, 2012). Humic materials are the most biological active compounds in soil that could stimulate plant growth as phytohormones (Traversa et al., 2013; Savy et al., 2017), enhance ATPase activity, promote nutrient utilization of plant (Jannin et al., 2012; Canellas et al., 2015), and stimulate the growth of bacteria (Tikhonov et al., 2010). In a previous study, we revealed that the water-soluble humic materials (WSHM) that are produced by lignite biodegradation enhanced the growth, cell metabolism, and nutrient transport of *Bradyrhizobium liaoningense* CCBAU05525, as well as its nodulation with soybean (12∼26% increased yield in soybean grains) (Gao T.G. et al., 2015). Therefore, WSHM could be a potential fertilizer to improve the legume yield.

In the present study, we examined the effects of WSHM on the QS system and the symbiotic nitrogen fixation of *S. meliloti*, and revealed that WSHM enhance the growth and nitrogen fixation of *S. meliloti* by regulating the QS system. This is the first report showing the effects of WSHM on the QS system in *S. meliloti*. Finally, we identified QsrR as a novel repressor of *expR* in this bacterium.

## Materials and Methods

### WSHM, Bacterial Strains, and Plasmids

Water-soluble humic materials were extracted from lignite collected from a Coal Mine in Inner Mongolia of China according to previously described methods (Dong et al., 2006; Jiang et al., 2013). Briefly, the lignite powder was inoculated with *Penicillium* sp. P6 and *Bacillus* sp. Y7 and incubated at 28°C for 2 weeks. Biodegraded lignite was diluted tenfold in deionized water, stirred, and centrifuged three times at 9000 ×*g* for 15 min. The supernatant was filtered through Whatman No. 1 filter paper and the filtrated (WSHM) were dried at 40°C for about 72 h, weighed, and stored in a vacuum-dried chamber. In the WSHM, 68 aromatic, aliphatic, and nitrogen-based compounds were detected by tetramethyl ammonium hydroxide (TMAH)-py-GC/MS (Gao T.G. et al., 2015).

All the bacterial strains and plasmids used in this study are listed in Supplementary Table S1. Among them, *S. meliloti* 1021 is a native mutant in which the ORF of *expR* is interrupted by an insertion; while *S. meliloti* 8530 is a derivative of *S. meliloti* 1021 by excising this insertion spontaneously. Since most *S*. *meliloti* strains harbor a functional *expR* (Pellock et al., 2002), *S*. *meliloti* 8530 was considered as the wild type strain. *Agrobacterium tumefaciens* KYC55 (pJZ372, pJZ384, pJZ410) was used as a bioassay strain for ultrasensitive detection of AHL.

### Growth Response of *S. meliloti* 8530 to WSHM

*Sinorhizobium meliloti* 8530 was preincubated in 50 mL of YM broth (Gao T.G. et al., 2015) for 4 days at 28°C with shaking (150 rpm). The culture was used to inoculate 50 mL of either YM broth (control) or YM broth with 500 mg L^-1^ WSHM at a final OD_600_ of 0.01. The cultures were incubated at 28°C with shaking (150 rpm) for 5–6 days and samples were plated using serial dilutions every 12 h to evaluate the rhizobia cell density. This assay was performed thrice in triplicate.

### Response of AHL Production to WSHM

**FIGURE 2:**
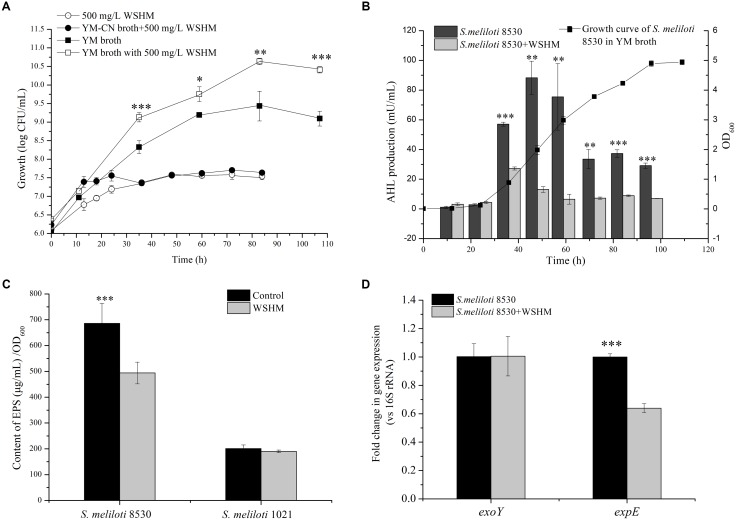
Effects of WSHM on the growth **(A)**, AHL production **(B)**, exopolysaccharide production **(C)**, and *exoY* and *expE* gene expression **(D)** in *S. meliloti* 8530. All the data are expressed as average ± standard deviation (SD) (*n* = 3). Statistical significance was assessed by Student’s *t*-tests (^∗^*P* < 0.05; ^∗∗^*P* < 0.01; ^∗∗∗^*P* < 0.001). For exopolysaccharides production **(C)**, *S. meliloti* 1021 (Δ*expR*) was also included. **(A)** YM-CN broth: the nitrogen (yeast extract) and carbon (mannitol) source in YM broth were removed.

*Agrobacterium tumefaciens* KYC55 (pJZ372, pJZ384, pJZ410) was incubated in AT medium [KH_2_PO_4_, 10.7 g; MgSO_4_ ⋅ 7H_2_O, 160 mg; CaCl_2_, 78 mg; FeSO_4_ ⋅ 7H_2_O, 5 mg; MnSO_4_ ⋅ H_2_O, 2.2 mg; (NH_4_)_2_SO_4_, 2 g; glucose, 5 g; in 1 L of distilled water; pH adjusted to 7.3 with K_2_HPO_4_; tetracycline, spectinomycin, and gentamycin at final concentrations of 2, 100, and 100 μg/mL respectively] with shaking at 150 rpm for 2 days to yield up to 10^9^ cells mL^-1^ (Zhu et al., 2003). The AHL production was detected as previously described (Zheng et al., 2006). Briefly, the cell-free supernatant of *S. meliloti* 1021 or 8530 culture (Figure 2B) was added to fresh AT broth at the ratio of 10% (v/v). The cell-free supernatant of *A. tumefaciens* strain R10 (pCF218) (Zhu et al., 2003) culture was used as positive control to assess the AHL sensitivity of *A. tumefaciens* KYC55 under these experimental conditions. The same volume of sterilized water (Control) or WSHM solution (500 mg L^-1^) was added separately into AT broth as negative controls. All the AT media prepared for tests were inoculated with approximately 10^7^ cells mL^-1^ of *A. tumefaciens* KYC55 and incubated with aeration (150 rpm) for 16–20 h at 28°C. The β-galactosidase activity in each culture was quantitatively analyzed to estimate the concentration of AHL (Zhu et al., 2003). β-galactosidase activity (Miller units) was calculated as OD_420_ × 10^3^/(time of reaction in minutes × volume of culture in milliliters × OD_600_) (Pérez-Montaño et al., 2011). Assays were conducted in triplicate for three times.

### Response of EPS Synthesis to WSHM Treatment

The effects of WSHM on EPS synthesis were analyzed for the wild type strain *S. meliloti* 8530, which can produce EPS I (succinoglycan), EPS II (galactoglucan), and linear mixed-linkage β-glucan (MLG) (Pérez-Mendoza et al., 2015), as well as for *S. meliloti* 1021, which could produce EPS I and extremely low level of EPS II (Pellock et al., 2002). Both strains were cultured in GMS medium (Staehelin et al., 2006) with and without 500 mg L^-1^ of WSHM for 5 days. EPSs were collected as described by Staehelin et al. (2006) and quantified in triplicate by the anthrone-sulfuric acid method (Jones, 2012).

### Expression of *exoY* and *expE* Genes in Response to WSHM

The effects of WSHM on the expression of *exoY* and *expE*, which are responsible for EPS I and EPS II synthesis, respectively (Mueller and González, 2010), were analyzed by reverse transcription quantitative real-time PCR (RT-qPCR). *S. meliloti* 8530 was cultured in YM broth (control) or YM broth supplied with 500 mg L^-1^ WSHM as mentioned above. Samples were collected at the end of exponential phrase (OD_600_ = 1.5–2.0). Total RNA was isolated using the RNA Pure Bacteria Kit (CWBIO, Beijing, China) according to the manufacturer’s instructions. First strand cDNAs were synthesized using PrimeScript Reverse Transcriptase (RT) (TaKaRa Code: D2680S) according to the manufacturer’s instructions. These cDNA samples were used for RT-qPCR with primers specific to *exoY* and *expE* genes (Supplementary Table S2). A 167 bp fragment of 16S rRNA gene was used as an internal control for normalization (Pérez-Montaño et al., 2014). Each 20 μL-reaction contained 10 μL of Power SYBR Green Master Mix (ABI, United States). The PCR program was: 95°C for 10 min, followed by; 40 cycles of 95°C for 15 s and 60°C for 1 min, followed by the melting curve. PCR was performed on an ABI 7500 Thermocycler and data were analyzed using the 2^-ΔΔCt^ method (Jannin et al., 2012). The experiment was performed for three times with four technical replicates.

### Expression of QS Genes in Response to WSHM

The bacteria were cultured same as described above (see Expression of *exoY* and *expE* Genes in Response to WSHM). The expression of QS genes following WSHM treatment was analyzed by RT-qPCR as mentioned above with primers specific to *sinI, sinR*, and *expR* (Supplementary Table S2). The cDNA obtained from cultures of *S. meliloti* 8530, *S. meliloti* 1021 (Δ*expR*), *S. meliloti* MG170 (Δ*sinR*), and *S. meliloti* MG32 (Δ*sinI*) (Supplementary Table S1) with or without WSHM were used as templates, and the experiment was performed three times with four replicates.

### Deletion of *qsrR* and Its Effect on *expR* Expression in Response to WSHM

*Agrobacterium tumefaciens* interacts with plant hosts similar to those of rhizobia. Thus, in order to analyze the repression mechanisms of WSHM on *expR* expression, the QS regulation system in *A. tumefaciens* (Gonzalez and Keshavan, 2006) was compared with that in *S. meliloti* 8530. AccR of *A. tumefaciens* can counteract *traR* repression by directly binding with opines produced by the plant host. Since protein SMc03890 in strain 8530, which was renamed as QsrR in this study, was very similar to AccR (with 31% similarity of amino acid sequence) and were both DeoR family transcriptional regulators, we hypothesized that QsrR could be capable of mediating the effects of WSHM on *expR* expression. To test this hypothesis, the *S. meliloti*Δ*qsrR* mutant strain was constructed via homologous recombination. Furthermore, by fusing the *expR* promoter region with the *lacZ* structural gene, the change in *expR* expression caused by the deletion of *qsrR* was determined by the activity of β-galactosidase. Refer to Supplementary Methods for detailed description of the process.

Previous reports have evidenced that the eukaryotic hosts are capable of interfering with bacterial QS by producing molecular signals, like flavonoid (Kalia, 2013; Nievas et al., 2017), and flavonoid homologs have been detected in WSHM (Gao T.G. et al., 2015). In order to analyze whether WSHM function as a plant signal (like opines) to represses QS in *S. meliloti*, *expR* gene expression levels in *S. meliloti* 8530 or *S. meliloti*Δ*qsrR* following treatment with either WSHM (500 mg L^-1^) or alfalfa seed exudates (2%, v/v) were determined by measuring the activity of β-galactosidase (Chai et al., 2010). Alfalfa seed exudates were prepared according to Cai et al. (2009). Assays were conducted in triplicate and repeated three times.

### QsrR Purification and Electrophoretic Mobility Shift Assays (EMSAs)

The full-length ORF of *qsrR* was amplified using the primers *qsrR*281 and *qsrR*282 (Supplementary Table S2) and cloned into pET28a. The recombinant plasmid pET28a-*qsrR* was transformed into *Escherichia coli* Rosetta (DE3) and cultured in LB medium with 0.4 mM isopropyl-β-D-thiogalactoside (IPTG) for induction at 37°C. The QsrR protein with a His tag (His_6_-QsrR) was purified with Ni-loaded nitrilotriacetic acid (NTA) resin (GE Healthcare) from cultures of *E. coli* Rosetta (DE3) carrying the recombinant plasmid. Electrophoretic mobility shift assays (EMSAs) were used to detect the interaction between the QsrR protein and the *expR* promoter using a DIG Gel Shift Kit, 2nd Generation (Roche), according to the manufacturer’s instructions. The promoter region of *expR* was amplified with primers *expR*11 and *expR*12 (Supplementary Table S2). Binding specificity was evaluated through addition of ∼100-fold excess of unlabeled *expR* promoter fragments, which competed with the labeled probe to bind with His_6_-QsrR. A labeled non-specific DNA probe from *Streptomyces avermitilis* was used as negative control. EMSAs were repeated at least twice.

### Bacterial One-Hybrid Assay

The experimental procedure was similar to that of Luo et al. (2014). Briefly, the gene *qsrR* was amplified with primers 03890BHf and 03890BHr (Supplementary Table S2), excised with Not I/Bgl II, and cloned into the bait plasmid pB1H1 to generate pB1H1-*qsrR*. Fragments R1 and R4 in the promoter region of *expR* were cloned into the prey plasmid pH3U3, respectively. pH3U3-R1 and pH3U3-R4 were not self-activating prey confirmed previously (see Supplementary Methods for detail). Then the plasmid pairs pB1H1-*qsrR*/pH3U3-R1 and pB1H1-*qsrR*/pH3U3-R4 were transformed into *E. coli* USO respectively. The growth of the transformants of *E. coli* USO, including USO: pB1H1-*zif268*/pH3U3-*zif268* as positive control (+/+); USO: pB1H1/pH3U3 (-/-), USO: pB1H1/pH3U3-R1 and USO: pB1H1/pH3U3-R4 as negative controls (-/+); and two transformants with *qsrR*, USO: pB1H1-*qsrR*/pH3U3-R1 and USO: pB1H1-*qsrR*/pH3U3-R4 (+/+), were determined on NM medium without histidine and with varying concentrations of 3-AT. We expected the transformants with *qsrR* to survive on NM medium containing 3-AT, as long as QsrR interacts with the *expR* promoter region to recruit RNA polymerase and activate the transcription of the reporter gene *HIS3*. Higher levels of *HIS3* gene expression enable the bacteria grow on NM medium with higher concentrations of 3-AT. Assays were repeated three times.

### Effects of WSHM on Plant Growth and Nodulation by *S. meliloti*

*Sinorhizobium meliloti* was cultured aerobically at 28°C in YM broth (Gao T.G. et al., 2015) for 2 days to OD_600_ = 1.0 (about 10^8^ CFU mL^-1^) and were used as inoculant. *Medicago sativa* seeds were surface-sterilized by 3% (v/v) NaClO for 3 min, germinated on 0.7% agar-water plates in the dark at 28°C for 24–48 h. The germinated seeds were planted in pots (three seeds per pot) filled with 300 cm^3^ of vermiculite and moisturized with low-N nutrient solution (Vincent, 1970). Six treatments with seven replicates (i.e., pots) were included: no inoculation control with and without WSHM (500 mg L^-1^); inoculation treatments of wild type strain (*S. meliloti* 8530, 1 mL) with and without WSHM; and inoculation treatments of Δ*qsrR* strain (1 mL) with and without WSHM (Table 1). Plants were grown in a greenhouse at 25 ± 2°C during the day and 17 ± 2°C at night with 60% relative humidity. Pots were rearranged daily to give a random distribution of growth conditions. After 45 days, all of the alfalfa plants were harvested and the number, fresh weight, and nitrogenase activity of the nodules, as well as the dry weight of the plants, were determined for each pot. The nitrogenase activity was analyzed by acetylene reduction assay, for which the whole roots with nodules in each pot (three plants) were put into a sealed bottle and incubated with acetylene (Suganuma et al., 1998), and the nitrogenase activity was calculated as μmol C_2_H_4_ /h ⋅ g of nodule. Then, the number and fresh weight of nodules in each pot were counted. The pooled data for each pot (three plants) were considered as one sample, and statistical analysis was conducted using Duncan test. Finally, several root nodules were sliced and treated for transmission electron microscopy (TEM) according to Bourassa et al. (2017) to evaluate the effects of WSHM treatment on the number and morphology of bacteroids in nodules induced by *S. meliloti* 8530.

**Table 1:** Effects of WSHM on nodulation of *M. sativa* inoculated with *S. meliloti*Δ*qsrR* or *S. meliloti* 8530 (WT) in the greenhouse^∗^.

Treatment	Nodule			Plant dry weight (mg/pot)
		
	Number of nodules	Fresh weight of	Nitrogenase activity	
	per pot^#^	nodule (mg/pot)	(μmol C_2_H_4_ /h ⋅ g)	
Control	0	0	0	46.4 ± 6.3a
WSHM	0	0	0	48.9 ± 10.1a
WT	21.75 ± 2.99ab	16.86 ± 3.35a	11.75 ± 2.56a	83.0 ± 7.0b
WT+WSHM	24.60 ± 2.30b	21.90 ± 5.03a	18.38 ± 5.64b	158.0 ± 26.0c
Δ*qsrR*	24.75 ± 7.14ab	20.24 ± 8.08a	19.92 ± 4.55b	87.0 ± 49.0b
Δ*qsrR*+WSHM	16.40 ± 3.51a	21.37 ± 7.28a	17.19 ± 3.84ab	98.0 ± 11.0b

*^∗^Values are expressed as average ± standard deviation (n = 7). Values in the same column that have different letters are statistically significant as assessed by Duncan test (P < 0.05). ^#^Three plants were included in each pot.*

## Results

### The Growth of *S. meliloti* in Response to WSHM

As shown in Figure 2A, the cell density of *S. meliloti* 8530 was about 3.5 × 10^7^ CFU/mL (83 h) in the WSHM solution or in YM-CN broth (without carbon/nitrogen source) supplied with WSHM. While it was 2.51 × 10^9^ CFU/mL in YM broth and 3.98 × 10^10^ CFU/mL in YM broth supplied with WSHM, which corresponds to 14.8-fold increase in cell density by WSHM at the stationary phase (Figure 2A). We could calculate from these data that only 0.09% of the total cell number increased in YM broth supplied with WSHM could be attributed to the additional nutrient sources from WSHM. Therefore, this growth enhancement can be mainly attributed to the stimulation effect of WSHM.

### Synthesis of AHL in Response to WSHM

In this analysis, the AHL detector strain, *A. tumefaciens* KYC55 did not respond to WSHM but did respond to the AHL produced by *A. tumefaciens* R10 (Supplementary Figure S1). Thus, *A. tumefaciens* KYC55 was used to investigate the effects of WSHM on AHL production in *S. meliloti*. AHL synthesis in *S. meliloti* 8530 started in the beginning of exponential phase and reached a peak during the middle of exponential phase (50 h); while the AHL synthesis during the whole potential phase was significantly decreased (60–93%) by WSHM (Figure 2B). WSHM treatment also reduced the production of AHL in *S. meliloti* 1021 by 57.9% (from 65 to 28 mU/mL, 50 h of incubation) (Supplementary Figure S1). Additionally, increased production of AHL by *A. tumefaciens* R10 in response to WSHM was observed (Supplementary Figure S1). These results suggest that WSHM could regulate the QS system in *S. meliloti*.

### EPS Synthesis and *exoY*/*expE* Expression of *S. meliloti* in Response to WSHM

Exopolysaccharides synthesis in *S. meliloti* 1021 was low (<200 μg ml^-1^) and was not affected by WSHM (Figure 2C); while EPS synthesis in *S. meliloti* 8530 was high (680 μg ml^-1^) without WSHM and was decreased by 30.87% (480 μg ml^-1^) with WSHM treatment.

For *expE*, a gene involved in EPS II synthesis and regulated by AHL-ExpR, its expression was down-regulated significantly by treatment with WSHM (Figure 2D); meanwhile, the expression of *exoY*, a gene involved in EPS I synthesis, was not affected by WSHM in *S. meliloti* 8530. All of these results suggest that WSHM did not affect the synthesis of EPS I, but decreased the production of EPS II and MLG.

### Impact of WSHM on the Expression of Genes Involved in QS

**FIGURE 3:**
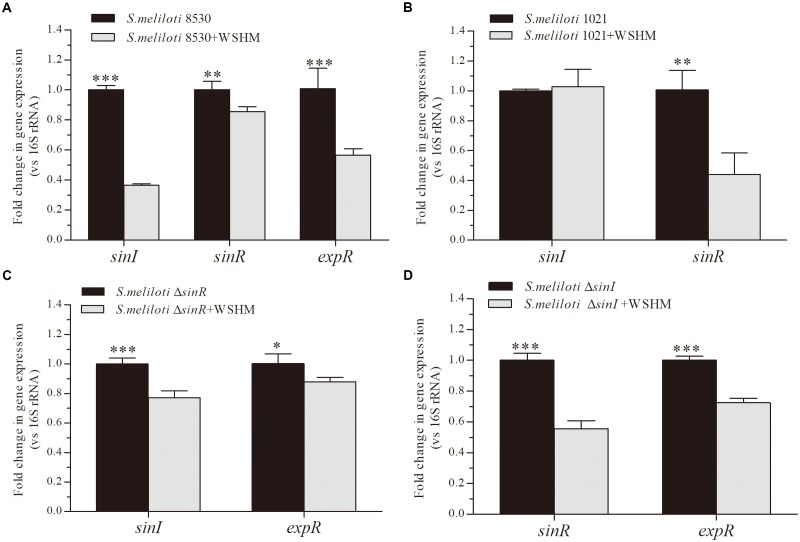
Effects of WSHM on the expression of genes involved in the QS system in *S. meliloti* 8530 **(A)**, *S. meliloti* 1021 **(B)**, *S. meliloti*Δ*sinR* **(C)**, and *S. meliloti*Δ*sinI* **(D)**. Data are expressed as average ± SD (*n* = 3). Statistical significance was determined using Student’s *t*-tests (^∗^*P* < 0.05; ^∗∗^*P* < 0.01; ^∗∗∗^*P* < 0.001).

In this analysis, WSHM treatment significantly down-regulated the expression of *sinI, sinR*, and *expR* in *S. meliloti* 8530 (Figure 3A); while in the *expR* mutant strain, *S. meliloti* 1021, WSHM only down-regulated *sinR* expression and did not alternate *sinI* expression (Figure 3B). In addition, the expression levels of both *sinI* and *expR* were down-regulated by WSHM in the *sinR* mutant (MG170, Figure 3C). These results suggest that the repression of *sinI* in *S. meliloti* 8530 by WSHM is due to the repression of *expR* and is independent of *sinR*. In the *sinI* mutant (MG32), the expression levels of the *sinR* and *expR* genes were down-regulated by WSHM (Figure 3D); suggesting that the effects of WSHM on *sinR* and *expR* expression are independent of *sinI*. Furthermore, the *sinR* expression was down-regulated by WSHM in both *S. meliloti* 8530 and *S. meliloti* 1021 (Δ*expR*), suggesting that the repression of *sinR* expression by WSHM is independent of ExpR.

### Effects of WSHM on the Expression of *expR* in *S. meliloti* Δ*qsrR*

**FIGURE 4:**
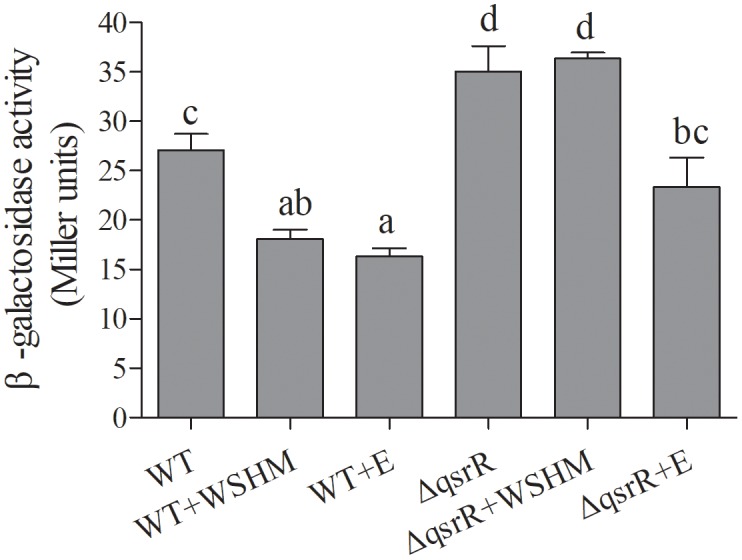
Expression of P*_expR_*-*lacZ* in *S. meliloti* 8530 (WT) and *S. meliloti*Δ*qsrR* mutant treated with either 500 mg L^-1^ WSHM or 2% alfalfa seed exudates (E), respectively. Alfalfa seed exudates were used to assess whether WSHM could repress *expR* expression in a manner similar with plant signal. Data are expressed as average ± SEM for three replicates. Different letters indicate statistical significance as assessed by Duncan tests (*P* < 0.05).

The expression level of the *expR* gene in *S. meliloti*Δ*qsrR* (SMc03890 deletion, see Section “Deletion of *qsrR* and Its Effect on *expR* Expression in Response to WSHM” for detail) was significantly higher than that in the WT (Figure 4), suggesting that QsrR represses *expR* transcription. Thus, the SMc03890 gene was renamed *qsrR*, for “quorum sensing regulator *expR* repressor.” As expected, the *expR* gene was down-regulated significantly in response to WSHM in *S. meliloti* 8530, but not in *S. meliloti*Δ*qsrR*, suggesting that QsrR is responsible for repressing *expR* expression in response to WSHM. Meanwhile, alfalfa seed exudates significantly repressed *expR* expression in both *S. meliloti* 8530 and *S. meliloti*Δ*qsrR* (Figure 4), suggesting that the repression of *expR* expression by seed exudates is independent of QsrR.

### Potentiated Interaction Between QsrR and *expR* Promoter by WSHM

**FIGURE 5:**
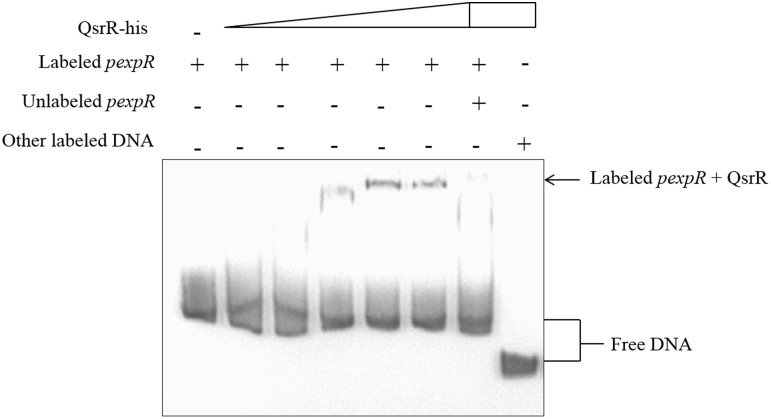
Electrophoretic mobility shift assays confirmed direct binding of QsrR to the *expR* promoter (*pexpR*). *pexpR* is a 286 bp DNA fragment from the translational start codon of gene *expR*. Each lane contained 0.005 nM of labeled *pexpR*. The labeled *pexpR* and a ∼100-fold excess of the unlabeled *pexpR* were used in competitive assays. Labeled non-specific DNA from *Streptomyces avermitilis* was used as negative control. The amount of His_6_-QsrR added in each lane were 0 μM, 0.92 Mm, 1.65 μM, 2.38 μM, 3.3 μM, 4.77 μM, 4.77 μM, and 4.77 μM, respectively.

**FIGURE 6:**
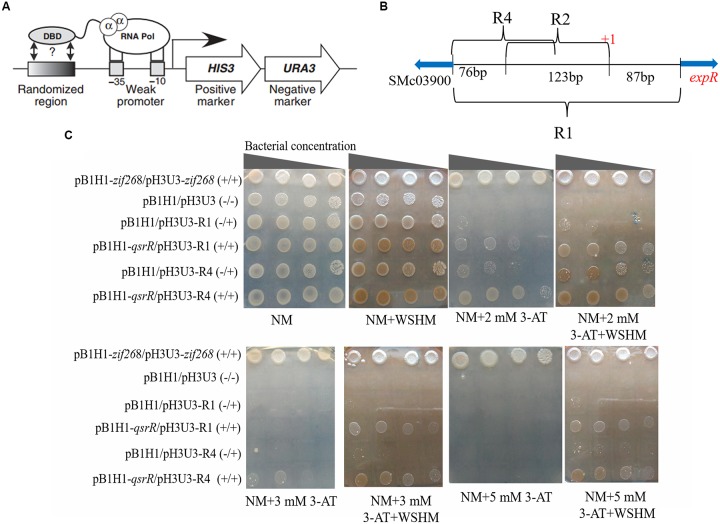
Bacterial-one-hybrid assays showing interaction between *expR* promoter (R1, R4) and QsrR which was potentiated by WSHM. **(A)** Schematic diagram of bacterial one-hybrid system. The HIS3/URA3 cistron is in the pH3U3 prey vector. The target DNA sequences were ligated to pH3U3. The binding of DNA-binding domain (DBD, ligated to pB1H1) with the target DNA sequence enables the transcription of *HIS3* and *URA3*. The expression of *HIS3* enabled *E. coli* USO grow on NM selective medium containing the desired concentration of 3-amino-triazole (3-AT), a competitive inhibitor of HIS3. The expression of *URA3* prevented *E. coli* USO growth on medium containing 5- fluoroorotic acid (5-FOA). This figure was adopted from Meng et al. (2005). **(B)** Schematic diagram of the promoter region of gene *expR* which was cloned into pH3U3. “+1” in red was marked as the transcription start site of *expR* which was located in 87 bp upstream of ATG of *expR* (Charoenpanich et al., 2013). Regions R1, R2, and R4 were ligated to pH3U3 prey vector to detect the binding region of QsrR. Region R1 is the entire intergenic region between *expR* and SMc03900. Region R2 is the middle region of R1 which lost the 87bp right flank and the 76bp left flank of R1. Region R4 is the left flank of R1 (142 bp). The blue arrows indicate transcriptional direction of *expR* and SMc03900. Region R2 was excluded from further analyses because of the high level self-activation of (*E. coli* USO: pB1H1/pH3U3-R2) (see Materials and Methods for details). **(C)** The interaction of QsrR and *pexpR* was assayed by the growth of bacteria on selective medium (NM lacking histidine) supplemented with 2, 3, or 5 mM of 3-AT. The genetic information of test strains is labeled on the left. “+/–” indicated that *qsrR* gene was cloned into pB1H1 (+) or the plasmid was left empty (–), and similar information for pH3U3. Images were assembled and the marked lines were eliminated without modifying the observed data in Adobe Photoshop CS5.

In order to test whether QsrR regulate the expression of *expR* directly or indirectly, EMSA were performed. As shown in Figure 5, a retarded DNA was observed when His_6_-QsrR (3.3 μM) was added to the assay mixture, indicating that QsrR was able to bind directly with the promoter region of *expR* gene. There was no retardation in the binding specificity assay or the negative control. These findings indicate that the transcription of *expR* is directly regulated by QsrR. In the EMSA test that determined the effect of WSHM on the interaction between QsrR and *expR* promoter, no band was detected when WSHM was added into the reaction system (data not shown). This might result from the possibility that WSHM interfered with the experimental process of EMSA, such as electrophoresis or binding of DNA with nylon membrane. Therefore, the role of WSHM in regulating *expR* expression via QsrR was further investigated by a bacterial one-hybrid assay (see Supplementary Methods and Supplementary Figure S3 for detail). The negative control strains *E. coli* USO (pB1H1/pH3U3-R1) and USO (pB1H1/pH3U3-R4) did not express *qsrR* and grew poorly; meanwhile, the positive strains USO (pB1H1-*qsrR*/pH3U3-R1) and USO (pB1H1-*qsrR*/pH3U3-R4) expressed *qsrR* and grew much better than the negative controls on selective medium (NM+3-AT) (Figure 6C). The positive strains were able to grow on medium with 2 mM of 3-AT that confirmed the ability of QsrR to bind with the promoter region of the *expR* gene. The binding region of QsrR is closer to the start of SMc03900 gene rather than that of *expR* (region R4, Figure 6B). In addition, the growth of the positive strains was significantly enhanced when WSHM were added to the selective medium; tolerance to 3-AT also increased from 2 to 5 mM. Meanwhile, growth of the negative control strains (USO: pB1H1/pH3U3-R1 and USO: pB1H1/pH3U3-R4) was not enhanced by WSHM on NM medium containing either 3 or 5 mM of 3-AT, further confirming that WSHM do not serve as a source of nutrients for these strains. These results suggest that QsrR can bind with the promoter region of *expR* gene, while WSHM can significantly potentiate this interaction.

### Effects of WSHM on *M. sativa* Growth and Nodulation With *S. meliloti* 8530

Water-soluble humic materials treatment increased plant dry weight by 5.4% compared to the control (Table 1). The nitrogenase activity and plant dry weight of *M. sativa* inoculated with *S. meliloti* 8530 and 500 mg L^-1^ of WSHM were significantly increased by 56.4 and 90.36%, respectively, compared with plants inoculated only with *S. meliloti* 8530, while no statistically significant difference was detected in nodule number and nodule fresh weight (13.1 and 29.9%) between these two treatments. No significant difference was observed in nitrogenase activity, plant dry weight, nodule number, or nodule fresh weight between the *M. sativa* inoculated with *S. meliloti*Δ*qsrR* +/- WSHM. Besides, the nitrogenase activity of plants inoculated with *S. meliloti*Δ*qsrR* alone was significantly increased by 69.53% compared with plants inoculated with *S. meliloti* 8530.

Transmission electron microscopy was performed to tested whether the increased nitrogenase activity of *M. sativa* inoculated with *S. meliloti* 8530 by WSHM was due to increased bacteroid density in nodules. The TEM images revealed that the number and morphology of bacteroids in alfalfa nodules induced by *S. meliloti* 8530 were not impacted by WSHM treatment (Supplementary Figure S4). Thus, WSHM may have affected the nitrogenase activity of the root nodules by other mechanisms, such as regulating the expression of nitrogen fixation genes or improving the energy supply to the bacteroids.

## Discussion

Quorum sensing regulates metabolically costly cooperative behaviors of bacteria depending on the environmental and physiological characteristics, such as production of exopolysaccharides, motility, and life-style switches related to symbiosis with eukaryotic hosts (Hense and Schuster, 2015; Calatrava-Morales et al., 2018). Many plant-associated bacteria communicate with the plant host through QS (González and Venturi, 2013; Schikora et al., 2016). The ExpR/Sin system is the sole QS system in *S. meliloti* 8530 (Krol and Becker, 2014) (Figure 1) that is involved in the regulation of metabolic and symbiotic procedures (Hoang et al., 2004; Gurich and Gonzalez, 2009). Our study regarding the effects of WSHM on the ExpR/Sin system in *S. meliloti* 8530 yielded several important findings.

SMc03890, renamed as *qsrR*, was shown to code a protein that directly represses *expR* gene transcription (Figures 4–6). As a versatile LuxR homolog regulator, ExpR directly or indirectly regulates the expression of at least 570 genes and plays a central role in the QS network in *S. meliloti* (Hoang et al., 2004; Gurich and Gonzalez, 2009; Charoenpanich et al., 2013). Thus, *qsrR* might have an essential function in different processes of *S. meliloti*. Previously, few studies on the regulation of the *expR* gene have been performed. One previous study showed that L-canavanine can repress *expR* expression as an arginine analog (Keshavan et al., 2005); meanwhile, Gao M. et al. (2015) reported that the RNA-binding protein Hfq regulates *expR* post-transcriptionally at higher population densities. Our study described QsrR as a novel repressor for *expR* transcription (Figure 4) and revealed the physical interaction between QsrR and the *expR* promoter by EMSA (Figure 5) and one-hybrid assays (Figure 6C). In addition, the increased nitrogenase activity in the nodules formed by *S. meliloti*Δ*qsrR* compared with those formed by *S. meliloti* 8530 (Table 1) suggests that QsrR may also regulate the expression of other genes, including those involved in nitrogen fixation. The function of QsrR is worthy of further research due to the versatile role of ExpR in regulating the metabolism and nodulation of *S. meliloti.* For example, the specificity of DNA sequences that can bind with QsrR might be further studied in order to reveal details in the interaction between QsrR and *expR* promoter.

The regulation of QS system in *S. meliloti* by WSHM via repressing *expR* expression (Figures 2–4) was evidenced for the first time, although different biological effects of humic materials on plants (Jannin et al., 2012; Traversa et al., 2013; Canellas et al., 2015; Savy et al., 2017) and bacteria (Tikhonov et al., 2010) have been demonstrated. The results from Figures 4–6 demonstrated that QsrR may mediate the repression of *expR* expression by WSHM. It is possible that WSHM also repress *sinR* expression via additional mechanisms since QsrR cannot bind with the promoter region of *sinR* (Supplementary Figure S2). Moreover, our results suggest that WSHM function in a manner comparable to alfalfa seed exudates and may act as plant signal to repress *expR* gene expression in *S. meliloti* (Figure 4). Previous reports have demonstrated that plants are able to produce compounds that mimic or inhibit bacterial QS processes to promote their development of beneficial traits (Gao et al., 2003; Schikora et al., 2016; Nievas et al., 2017). WSHM might help plants to regulate bacterial QS and improve the symbiotic relationship between the host plant and bacteria. Compounds such as furan, pyrrole, benzpyrole, benzene rings, and esters have been identified in WSHM (Gao T.G. et al., 2015) and they may interfere with microbial QS systems (Gonzalez and Keshavan, 2006; Christensen et al., 2013). However, further investigation is needed to identify the compounds responsible for *S. meliloti* QS regulation in WSHM or alfalfa seed exudates.

In addition, the results suggest that the inactivation of rhizobia ExpR/Sin genes in nodules might be due to regulation by the plant host. Even though the QS system in *S. meliloti* has been reported to control cell functions essential for successful plant invasion, the ExpR/Sin genes were inactive in nodules due to an unknown mechanism (Gurich and Gonzalez, 2009). Alfalfa seed exudates and WSHM repressed the expression of *expR* in *S. meliloti* (Figure 4), suggesting that the inactivity of ExpR/Sin genes in nodules is a response of rhizobia to the host signal molecules. The repression of *expR* by host signal might due to the presence of L-canavanine in alfalfa seed exudates and L-canavanine could cause misfolding of the ExpR protein (Keshavan et al., 2005), while ExpR has been reported to possess the potential for self-regulation (Charoenpanich et al., 2013).

Water-soluble humic materials can stimulate *S. meliloti* growth by repressing QS. Although WSHM contain 52.18% C and 3.72% N (Gao et al., 2012) and humic acids could act as C or N source for bacteria growth (Tikhonov et al., 2010; Gao T.G. et al., 2015), only 0.09% of the increased biomass in WSHM treatment could be contributed to the C/N supply by WSHM in YM broth (Figure 2A). Thus, we conclude that WSHM increase *S. meliloti* 8530 growth mainly due to the regulatory effects on cell metabolism. The repression of the QS system in *S. meliloti* 8530 by WSHM could stimulate growth (Figures 2, 3), which supported the observation that QS restrains growth in *S. meliloti, M. huakuii, R. leguminosarum*, and *Rhizobium* sp. NGR234 (Wilkinson et al., 2002; He et al., 2003; Gao et al., 2006; Charoenpanich et al., 2015).

Water-soluble humic materials could regulate EPS synthesis, mainly decrease EPS II and MLG production, but do not affect EPS I production (Figures 2C,D). Amongst the three kinds of EPS produced by *S. meliloti* 8530 (Pérez-Mendoza et al., 2015), EPS I is the most efficient compound at initiating and maintaining infection threads (Jones, 2012). The probability of causing aberrant infection threads by EPS II, which is less efficient than EPS I at inducing infection thread formation, is 8–10 times higher than that of EPS I (Pellock et al., 2000). Furthermore, EPS synthesis in rhizobia is energy consuming; thus, EPS II is not required if EPS I is present (Zhan et al., 1989; Mithöfer, 2002). Even though MLG is involved in the attachment of *S. meliloti* to alfalfa roots, there was no significant difference in nodulation occupancy between a wild-type and a MLG synthesis deficient mutant (Pérez-Mendoza et al., 2015). Therefore, EPS II and MLG are not likely to be essential for efficient nodulation between *S. meliloti* and alfalfa. This notion is supported by the fact that the nodule occupancy of *S. meliloti* 1021 (Δ*expR*) was 10–20% higher than that of *S. meliloti* 8530 (Charoenpanich et al., 2015). Thus, shutting down EPS II and MLG production with WSHM may promote symbiotic nitrogen fixation between *S. meliloti* and *M. sativa.* It is noteworthy that the ExpR/Sin QS system can regulate EPS II and MLG production (Mueller and González, 2010; Pérez-Mendoza et al., 2015), while EPS I biosynthesis becomes independent of QS in the absence of ExpR, and mutations to *expR* did not alter the amounts of EPS I produced (Glenn et al., 2007). This may be because MucR increases the production of EPS I independently of QS (Mueller and González, 2010). Thus, WSHM may regulate EPS production in *S. meliloti* 8530 through their regulation of the QS system.

Water-soluble humic materials improved symbiotic nitrogen fixation between *M. sativa* and *S. meliloti* (Table 1). Since WSHM did not affect the density of bacteroids in nodules (Supplementary Figure S4), the increase in nitrogenase activity of *M. sativa* following treatment with WSHM (Table 1) may be due to the enhanced expression of *nifA* gene via repressing *expR* expression. It has been reported that AHL-ExpR repress the expression of genes related to nitrogen fixation, such as *fixTQK*, which induce the expression of *nifA* (Hoang et al., 2004; Charoenpanich et al., 2013). In addition, the failed of significant increase in nodule number and nodule fresh weight of *M. sativa* by WSHM may result from the fact that nodule number and nodule weight are controlled by the plant host to ensure optimal growth (Mortier et al., 2012; Zipfel and Oldroyd, 2017). The promotion of nitrogen fixation between *S. meliloti* and *M. sativa* by WSHM offers an economical and efficient route for improving biological nitrogen fixation in agriculture. In a field experiment, WSHM treatment (500 mg/mL) on seeds (375 g WSHM per ha) increased alfalfa yield by 29% per year (unpublished data).

## Conclusion

We identified QsrR as a direct repressor of *expR* (a gene central to the QS system in *S. meliloti* 8530) transcription. WSHM were proposed to repress *expR* expression by modulating the interaction between QsrR and the *expR* promoter, and ultimately decrease AHL, EPS II and MLG production. Consequently, WSHM increased the growth of *S. meliloti*, as well as symbiotic nitrogen fixation with *M. sativa*. In addition, both *expR* and *sinR* were down-regulated by WSHM with independent mechanisms. We hypothesize that QsrR may mediate the repression of *expR* expression by WSHM; however, the mechanism by which WSHM down regulate *sinR* expression requires further investigation.

## Author Contributions

Y-YX, J-SY, CL, and H-LY conceived and designed the study. Y-YX, R-NW, and X-QQ performed the experiments. Y-YX and E-TW wrote the manuscript. Y-YX, and B-ZL participated in the preparation of water-soluble humic materials. H-LY and W-FC helped to design the experiments and drafted the manuscript.

## Conflict of Interest Statement

The authors declare that the research was conducted in the absence of any commercial or financial relationships that could be construed as a potential conflict of interest.
