# Supplementary material for: Water-Soluble Humic Materials Regulate Quorum Sensing in Sinorhizobium meliloti Through a Novel Repressor of expR
Source: Front Microbiol. 2018 Dec 21;9:3194. doi: 10.3389/fmicb.2018.03194 (PMC6309736; doi:10.3389/fmicb.2018.03194)
Supplement: Supplementary file 1 [file Data_Sheet_1.docx]

Supplementary Material

**Water-soluble Humic Materials Regulate Quorum Sensing in *Sinorhizobium meliloti* Through a Novel Repressor of *expR***

**Yuanyuan Xu^1^, Jinshui Yang****^1^, Cong Liu^1^, Entao Wang^2^, Ruonan Wang^1^, Xiaoqian Qiu^1^, Baozhen Li^1^, Wenfeng Chen^1^, Hongli Yuan^1*^**

^1^State Key Laboratory of Agrobiotechnology and Key Laboratory of Soil Microbial, Ministry of Agriculture, College of Biological Sciences, China Agricultural University, Beijing, P. R. China

^2^Escuela Nacional de Ciencias Biológicas, Instituto Politécnico Nacional, México, Mexico

^*^**Corresponding**:

Corresponding author. hlyuan@cau.edu.cn (H. L. Yuan)

1. **Supplementary Methods**
   1. ***S. meliloti* Δ*qsrR* mutant construction via homologous recombination**

DNA sequences upstream and downstream of the *qsrR* gene were amplified from genomic DNA of *S. meliloti* 8530 with the primer pairs 03890up1/03890up3 and 03890down1/03890down2 (Supplementary Table S2), respectively. The two PCR fragments were digested with the restriction endonuclease pair Xba I/Not I or Apa I/Age I, respectively, and then ligated to the suicide vector pCM351 (Marx and Lidstrom, 2002) to generate pCM351-up-down. pCM351-up-down was inserted into *S. meliloti* 8530 with the helper plasmid pRK2013. Potential double recombinants were selected based on gentamicin and spectinomycin resistance and tetracycline sensitivity. The *qsrR* deletion was confirmed by PCR with the primer pairs 03890yanup1/03890yanup2; 03890yandown1/03890yandown2; 03890yanup1/GUR, and; GDF/03890yandown2, followed by sequencing with the primers 03890ceyan1/03890ceyan2 (Supplementary Table S2).

To determine the change in *expR* expression caused by the deletion of *qsrR,* the promoter region of *expR* was fused with the *lacZ* structural gene in a plasmid, therefore, the expression level of *expR* was linked to the activity of β-galactosidase. The promoter region of the *expR* gene was amplified from genomic DNA of *S. meliloti* 8530 using the p*exp*TT3 and p*exp*TT4 primers (Supplementary Table S2); then, amplified DNA was ligated into pPR9TT, a low copy-number *lacZ*-based promoter probe plasmid (Santos et al., 2001). This recombinant plasmid (pPR9TT*-*p*expR*) was inserted into *S. meliloti* 8530 and *S. meliloti* Δ*qsrR* using a helper plasmid (pRK2013).

- 1. **Bacterial one-hybrid assay**

The bacterial one-hybrid (B1H) system (Meng et al., 2005) was used to determine the role of WSHM in the interaction between QsrR and the *expR* promoter. The protein sequences and target DNA were cloned into the bait plasmid pB1H1 and prey plasmid pH3U3 (Supplementary Fig. S3), respectively. These two plasmids were then co-transformed into *E. coli* USO (*hisB*^-^ *pyrF*^-^). *E. coli* USO (*hisB*^-^ *pyrF*^-^) has a deletion in both the *hisB* and *pyrF* genes (the bacterial homologs of *HIS3* and *URA3*) and histidine are essential for its growth. If the DNA-binding domain (DBD) bound with the target DNA, the fusion to the α-subunit recruits RNA polymerase to the weak *lac* promoter and activates transcription of *HIS3* and *URA3* (Fig. 6A). The expression of *HIS3* enables *E. coli* USO to grow on NM selective medium containing varying concentrations of 3-amino-triazole (3-AT), a competitive inhibitor of *HIS3* gene product. The higher concentration of 3-AT which *E. coli* USO can tolerate, the stronger interaction between the target protein and the DNA sequence. The Cys_2_His_2_ zinc finger protein, Zif268 and its binding site *zif268* were introduced into pB1H1 and pH3U3, respectively and were used as positive control in this B1H system (Meng et al., 2005).

To eliminate self-activating prey (i.e., *HIS3* expressed in the absence of the target protein) and locate the subregion for which QsrR has an affinity, three fragments in the promoter region of *expR* (Fig. 6B) were amplified with the primer pairs *expR*11/*expR*12, *expR*21/*expR*22, and *expR*11/*expR*42 (Supplementary Table S2). Fragment R1 is the entire intergenic region between *expR* and SMc03900. Fragment R2 is the middle region of R1. Fragment R4 is the left flank of R1 (142 bp) (Fig. 6B). The three PCR fragments were cloned into the prey plasmid pH3U3 to generate pH3U3-R1, pH3U3-R2, and pH3U3-R4. The plasmid pairs pB1H1/pH3U3-R1, pB1H1/pH3U3-R2, and pB1H1/pH3U3-R4 were then co-transformed into *E. coli* USO. The self-activation of transformants was determined on NM medium (Meng et al., 2005). The transformant with the R2 fragment (*E. coli* USO: pB1H1/pH3U3-R2) grew on NM medium containing 3 mM 3-AT, demonstrating its high-level self-activation, so it was excluded from further analyses. Meanwhile, the growth of the transformants with the R1 and R4 fragments (USO: pB1H1/pH3U3-R1 and USO: pB1H1/pH3U3-R4) on NM medium was completely inhibited by 2 mM 3-AT. Therefore, these two transformants were used to detect the interactions between QsrR and the *expR* promoter at 3-AT concentrations above 2 mM.

# Supplementary Figures and Tables

# Supplementary Tables

Supplementary Table S1. Bacterial strains and plasmids used in this study

| Strains or plasmids | Description | Reference | |
| --- | --- | --- | --- |
| Strains | | | |
| *S. meliloti* 1021 | *expR102*::IS*Rm*2011-1 | (Galibert et al., 2001) | |
| *S. meliloti* 8530 | 1021, *expR*^+^ | (Pellock et al., 2002) | |
| *S. meliloti* MG32 | 8530 Δ*sinI* | (Gao et al., 2005) | |
| *S. meliloti* MG170 | 8530 Δ*sinR* | (Gao et al., 2012) | |
| *Agrobacterium tumefaciens* KYC55(pJZ372)(pJZ384) (pJZ410) | AHL biosensor strain | (Zhu et al., 2003) | |
| *A. tumefaciens* R10 (pCF218) | TraR overexpressed in *A. tumefaciens* | (Zhu et al., 2003) | |
| *E. coli* US0 *hisB-pyrF*- | *E. coli XL-1* blue F' episome, *hisB*-, *pyrF-* | (Meng et al., 2005; Luo et al., 2014) | |
| Plasmids | | | |
| pB1H1 | Expression vector for Bacterial one-hybrid system, Cm^R^,  p15A_origin | | (Meng et al., 2005; Luo et al., 2014) |
| pB1H1-*qsrR* | Recombinant plasmid for *qsrR* expression | | This study |
| pH3U3-mcs | Reporter vector for Bacterial one-hybrid system, KmR, f1_origin | | (Meng et al., 2005; Luo et al., 2014) |
| pH3U3-R1 | Recombinant plasmid for expressing *expR* promoter R1 | | This study |
| pH3U3-R2 | Recombinant plasmid for expressing *expR* promoter R2 | | This study |
| pH3U3-R4 | Recombinant plasmid for expressing *expR* promoter R4 | | This study |
| pCM351 | Suicide plasmid | | (Lidstrom, 2002) |
| pCM351-up-down | Homologous recombination plasmid for *qsrR* gene knock out | | This study |
| pRK2013 | Conjugal transfer helper plasmid | | (Flgurski and Helinski, 1979) |
| pPR9TT | Promoter probe vector | | (Santos et al., 2001) |
| pPR9TT-p*expR* | Analisis the expression level of *expR* promoter | | This study |
| pET28a | Vector for protein expression | | ([Luo et al., 2014](#_ENREF_7)) |

Supplementary Table S2. Primers used in this study

| Primers | Sequence (5’ →3’) | | Size of PCR product (bp) | Reference |
| --- | --- | --- | --- | --- |
| Primers used in Q-PCR | |  |  |  |
| 16s rRNA forward  16s rRNA reverse | GGTGAGTAACGCGTGGGAAT  CAGCTATGGATCGTCGCCTT | | 167 | This study |
| *sinI* forward  *sinI* reverse | GGCATCAGCCATATCGTCA  ACCGCCTTCATCGTTTCG | | 167 | This study |
| *sinR* forward  *sinR* reverse | GGCTAATCAACAGGCTGTCCT  CAGGCGGCAGATTCTCAAA | | 163 | This study |
| *expR* forward  *expR* reverse | TTCGACTTCTACGGCATCGTG  TCGGATCGATAACGACGTATTTCT | | 130 | (Gurich and Gonzalez, 2009) |
| *exoY* forward  *exoY* reverse | GCTCCTCAACATCATTCG  TTCTGGACATATTGCGTGT | | 198 | This study |
| *expE* forward  *expE* reverse | GCCAAACACACGCTCGTCAT  GCCACTCTCCGCAAGAGAAA | | 75 | (Mueller and González, 2010) |
| Primers used in Bacterial-one hybrid assay | | | | |
| 03890BHf | ATGCGGCCGCAAGGAGATTCCAGGATGTCCACCGATCTGCTGCTG | | 783 | This study |
| 03890BHr | CGAAGATCTTCACCGGATAGGCTCCTTCGCGAGAACG | |  |  |
| *expR*11 | ATGCGGCCGCCCACCTTGCGTGCCGGCAAAG | | 286 | This study |
| *expR*12 | CGGAATTCAGAGCATCCTCAAATCAAAAACGCG | |  |  |
| *expR*21 | ATGCGGCCGCAGCGAGAAGGATCATCCGATAC | | 123 | This study |
| *expR*22 | CGGAATTCTAATTCTTCATATCACGCCCGG | |  |  |
| *expR*42 | CGGAATTCCATCAAGATAGCTAACGATGAATC | | 142 | This study |
| Primers used in *qsrR* mutant construction | | | | |
| 03890up1 | GCTCTAGACTTTCCGCACCGCAGACAATCG | | 1398 | This study |
| 03890up3 | TGGACATGCGGCCGCCCTGGAACCGTGCATAATCGT | |  |  |
| 03890down1 | TATGGGCCCATGTCGAAAAACCGGCTTGCCG | | 1400 | This study |
| 03890down2 | CGAACCGGTCCGGAGCACCGCCGCCGACAAT | |  |  |
| 03890yanup1 | GAAAGGCCGCGTCAAAGGCATCC | | 1713 bp for WT and none for Δ*qsrR* | This study |
| 03890yanup2 | CATAGACGCGCTCGCAAAGGCC | |  |  |
| 03890yandown1 | GCGGCTGATCGCTTCCAGGAGC | | 1961 bp for WT and none for Δ*qsrR* | This study |
| 03890yandown2 | GCCTCGGCGCAGGCCGGATGATC | |  |  |
| GUR | CGAATGATGCCCATACTTGAGCC | | With 03890yanup1: 1902 bp for Δ*qsrR* and none for WT | This study |
| GDF | GTTGGGCATACGGGAAGAAGTG | | With 03890yandown2: 1656 bp for Δ*qsrR* and none for WT | This study |
| 03890ceyan1 | TTTTTCCTCAGATGGCCGATTCCC | | 1421 bp for WT and 1558 bp for Δ*qsrR* | This study |
| 03890ceyan2 | GACTTCTCGACCTCGACGGCATTG | |  |  |
| p*exp*TT3 | CTTCATAGATCTCCACCTTGCGTGCCGGCAAAG | | 286 | This study |
| P*exp*TT4 | ATTCATAAGCTTAGAGCATCCTCAAATCAAAAAC | |  |  |
| Primers used in EMSA | | | | |
| *qsrR*281 | TTCCAGGGCTAGCATGTCCACCGATCTGCTGCT | | 783 | This study |
| *qsrR*282 | ATCGCGGAGGATCCTCACCGGATAGGCTCCTTCG | |  |  |

- 1. **Supplementary Figures**





**Supplementary Figure S1:** Bioassay of WSHM effects on AHL production of *S. meliloti* 1021 (50 h of incubation) represented by β-galactosidase activity of *A. tumefaciens* KYC55 (detector) and with *A. tumefaciens* R10 as positive control. **Control**: AHL content in sterilized water; **WSHM**: AHL content in WSHM solution of 500 mg L^-1^; **1021**: AHL production in supernatants of *S. meliloti* 1021; **1021+WSHM**: AHL production in supernatants of *S. meliloti* 1021 treated with 500 mg L^-1^ WSHM. **R10**: AHL production in supernatants of the positive control strain *A. tumefaciens* R10 which produces 3-O-C6-HSL and 3-O-C8-HSL. **R10+WSHM**: AHL production in supernatants of *A. tumefaciens* R10 treated with 500 mg L^-1^ WSHM. Bars with different letters indicates significant difference from each other according to Duncan test (*P*<0.05).


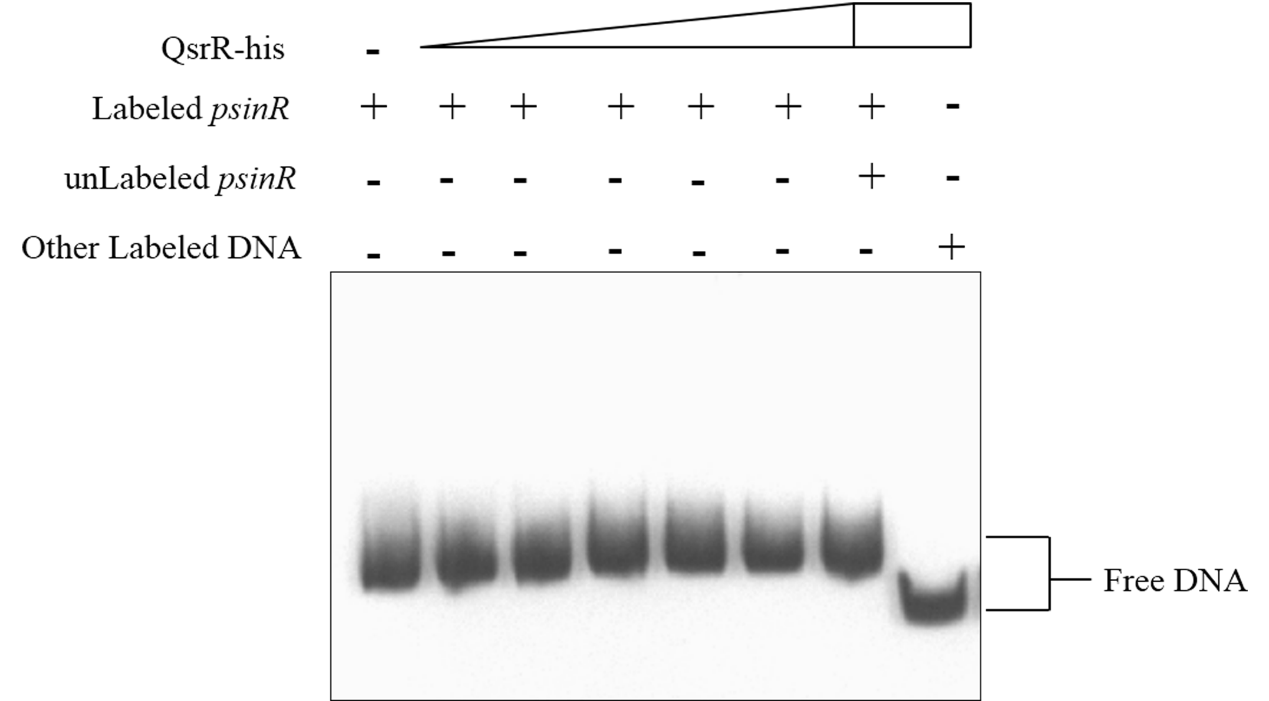


**Supplementary** **Figure S2.** EMSA confirmed no direct binding of QsrR to *sinR* promoter (*psinR*). *psinR:* a 205 bp DNA fragment from the translational start codon of gene *sinR*. Each lane contained 0.005 nM labeled *psinR*. The labeled *psinR* and a ~100-fold excess of the unlabeled *psinR* were used in competitive assays. Labeled nonspecific DNA from *Streptomyces coelicolor* was used as negative control. The amount of His_6_-QsrR added in each lane were 0 μg, 0.5 μg, 0.9 μg, 1.3 μg, 1.8 μg, 2.6 μg, 2.6 μg and 2.6 μg respectively. There was no observable shift suggesting that His_6_-QsrR does not bind with *psinR.*


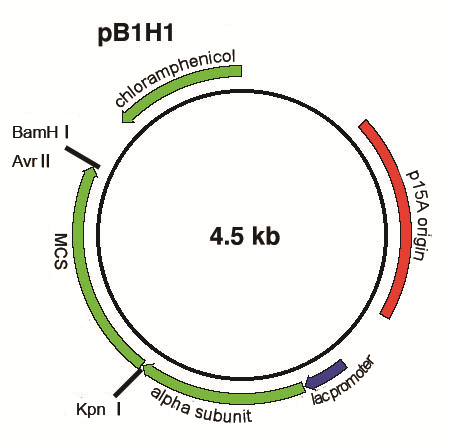


A


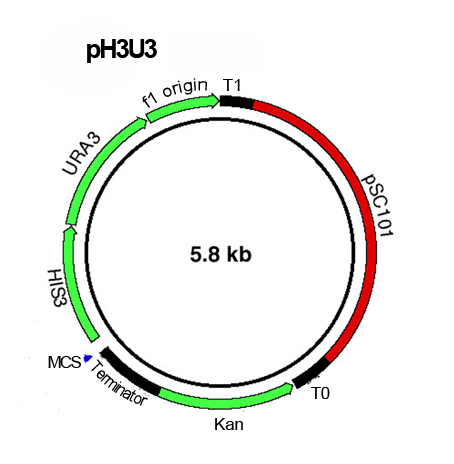


B

**Supplementary Figure S3.** Maps of plasmids pB1H1 (A) and pH3U3 (B) used in Bacterial one-hybrid system.


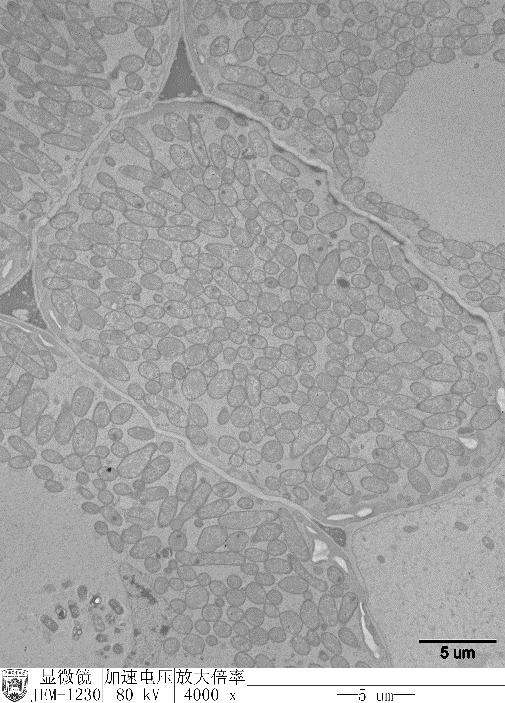


WT


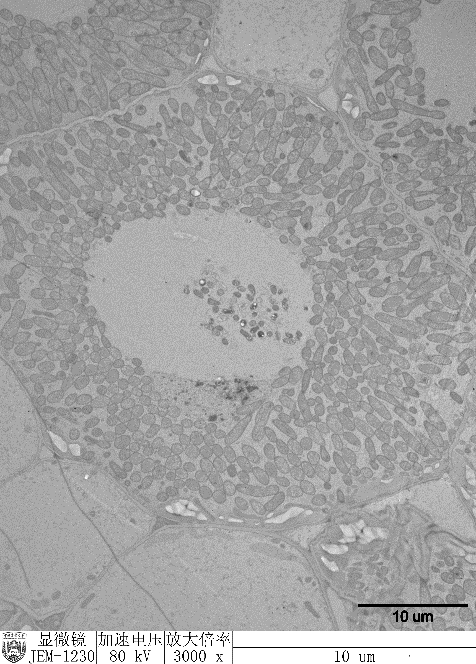


WT


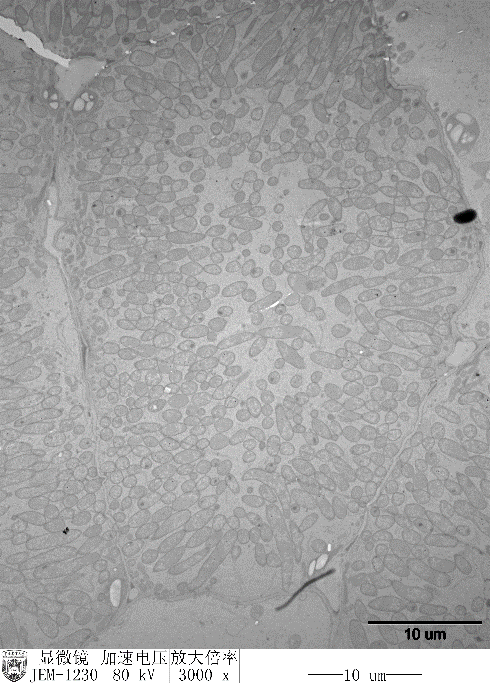


WT+WSHM

+


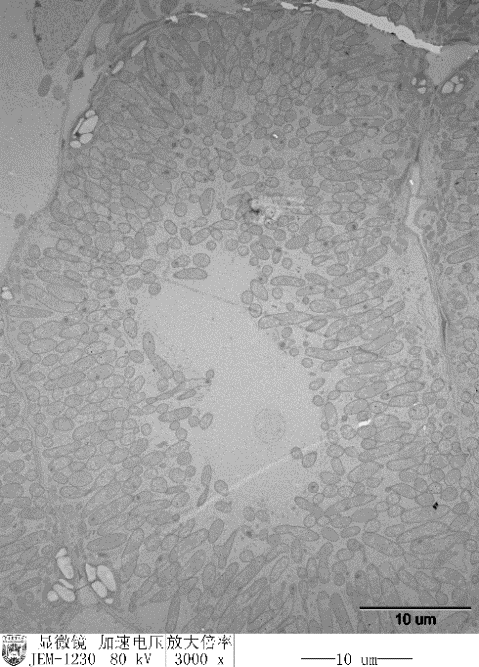


WT+WSHM

+


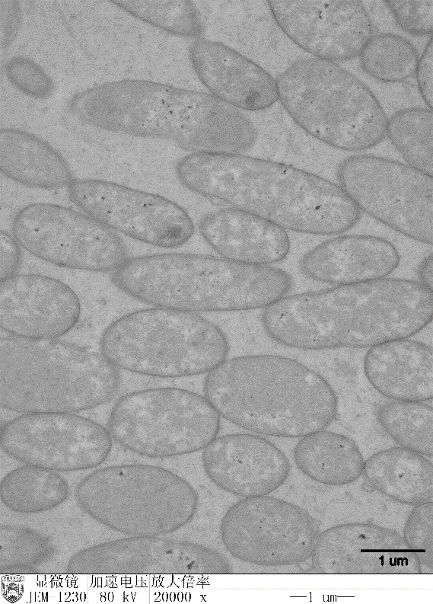


WT


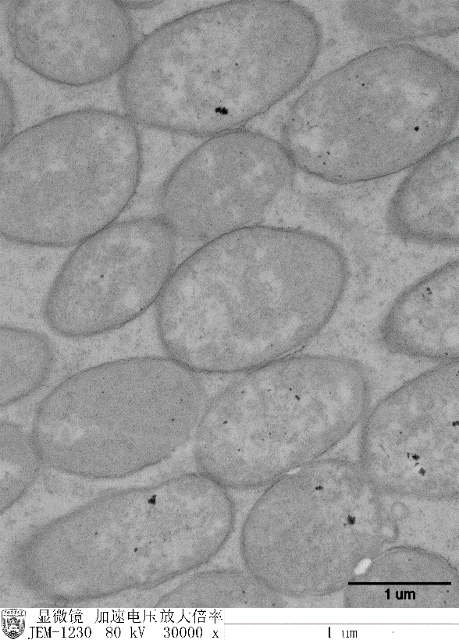


WT+WSHM

+

**Supplementary Figure S4.** The microstructures of nodules of *M. sativa* inoculated with *S. meliloti* 8530 treated with WSHM (WT+WSHM) or not (WT) analyzed by transmission electron microscope (TEM).

**Reference**

Charoenpanich, P., Meyer, S., Becker, A., and McIntosh, M. (2013). Temporal expression program of quorum sensing-based transcription regulation in *Sinorhizobium meliloti*. J Bacteriol 195, 3224-3236.

Flgurski, D.H., and Helinski, D.R. (1979). Replication of an origin-containing derivative of plasmid RK2 dependent on a plasmid function provided in trans. Proc. Nat.l Acad. Sci. U. S. A. 76, 1648-1652.

Galibert, F., Finan, T.M., Long, S.R., Pühler, A., Abola, P., Ampe, F. et al. (2001). The composite genome of the legume symbiont *Sinorhizobium meliloti*. Science 293, 668-672.

Gao, M., Coggin, A., Yagnik, K., and Teplitski, M. (2012). Role of specific quorum-sensing signals in the regulation of exopolysaccharide II production within *Sinorhizobium meliloti* spreading colonies. PLoS One 7, e42611.

Gao, M., Chen, H., Eberhard, A., Gronquist, M.R., Robinson, J.B., Rolfe, B.G., and Bauer, W.D. (2005). *sinI*- and *expR*-Dependent Quorum Sensing in *Sinorhizobium meliloti*. J. Bacteriol. 187, 7931-7944.

Gurich, N., and Gonzalez, J.E. (2009). Role of Quorum Sensing in *Sinorhizobium meliloti*-Alfalfa Symbiosis. J. Bacteriol. 191, 4372-4382.

Lidstrom, C.J.M.a.M.E. (2002). Broad-Host-Range cre-lox System for Antibiotic Marker Recycling in Gram-Negative Bacteria. BioTechniques 33, 1062-1067.

Luo, S., Sun, D., Zhu, J., Chen, Z., Wen, Y., and Li, J. (2014). An extracytoplasmic function sigma factor, sigma(25), differentially regulates avermectin and oligomycin biosynthesis in *Streptomyces avermitilis*. Appl. Microbiol. Biotechnol. 98, 7097-7112.

Marx, C.J., and Lidstrom, M.E. (2002). Broad-host-range cre-lox system for antibiotic marker recycling in gram-negative bacteria. Bio.Techniques. 33, 1062-1067.

Meng, X., Brodsky, M.H., and Wolfe, S.A. (2005). A bacterial one-hybrid system for determining the DNA-binding specificity of transcription factors. Nat. Biotechnol. 23, 988-994.

Mueller, K., and González, J.E. (2010). Complex Regulation of Symbiotic Functions Is Coordinated by MucR and Quorum Sensing in *Sinorhizobium meliloti*. J. Bacteriol. 193, 485-496.

Pellock, B.J., Teplitski, M., Boinay, R.P., Bauer, W.D., and Walker, G.C. (2002). A LuxR Homolog Controls Production of Symbiotically Active Extracellular Polysaccharide II by *Sinorhizobium meliloti*. J. Bacteriol. 184, 5067-5076.

Santos, P.M., Bartolo, I.D., Blatny, J.M., Zennaro, E., and Valla, S. (2001). New broad-host-range promoter probe vectors based on the plasmid RK2 replicon. FEMS Microbiol. Lett. 195, 91-96.

Zhu, J., Chai, Y., Zhong, Z., Li, S., and Winans, S.C. (2003). Agrobacterium Bioassay Strain for Ultrasensitive Detection of *N*-Acylhomoserine Lactone-Type Quorum-Sensing Molecules: Detection of Autoinducers in *Mesorhizobium huakuii*. Appl. Environ. Microbiol. 69, 6949-6953.
